# MIPs–SERS Sensor Based on Ag NPs Film for Selective Detection of Enrofloxacin in Food

**DOI:** 10.3390/bios13030330

**Published:** 2023-02-28

**Authors:** Jing Neng, Yazhi Wang, Yilong Zhang, Peng Chen, Kai Yang

**Affiliations:** 1 College of Food Science and Engineering, Zhejiang University of Technology, Huzhou 313299, China; 2 College of Computer Science and Engineering, Zhejiang University of Technology, Hangzhou 310027, China

**Keywords:** surface-enhanced Raman spectroscopy, molecularly imprinted polymers, biosensor, enrofloxacin

## Abstract

The quinolone antibiotics represented by enrofloxacin (ENRO) are harmful to the ecological environment and human health due to illegal excessive use, resulting in increasing food residues and ENRO levels in the environment. To this end, we developed a MIPs–SERS method using surface-enhanced Raman spectroscopy (SERS) and molecularly imprinted polymers (MIPs) to detect ENRO in food matrices. Firstly, a layer of silver nanoparticles (Ag NPs) with the best SERS effect was synthesized on the surface of copper rods as the enhancing material by in situ reductions, and then MIPs targeting ENRO were prepared by the native polymerization reaction, and the MIPs containing template molecules wrapped on the surface of silver nanoparticle films (Ag NPs–MIPs) were obtained. Our results showed that the Ag NPs–MIPs could specifically identify ENRO from the complex environment. The minimum detection limit for ENRO was 0.25 ng/mL, and the characteristic peak intensity of ENRO was linearly correlated to the concentration with a linear range of 0.001~0.1 μg/mL. The experimental results showed that in comparison to other detection methods, the rapid detection of ENRO in food matrices using Ag NPs–MIPs as the substrate is reliable and offers a cost-effective, time-saving, highly selective, and sensitive method for detecting ENRO residues in real food samples.

## 1. Introduction

Enrofloxacin (ENRO) is a chemically synthesized third-generation quinolone broad-spectrum antimicrobial drug [1], an extremely important class of antimicrobial moieties that are often used to treat skin infections, respiratory infections, and other diseases in animals, and are also added to feed and drinking water as preventive medicine for diseases. The use of antimicrobials such as ENRO has greatly improved the yield of livestock and aquaculture and reduced losses due to bacterial infections in the breeding process. However, because of its long half-life and slow metabolism, it can negatively affect human health through bioaccumulation and biomagnification [2]. Long-term ingestion of foods with excessive ENRO residues may cause adverse symptoms such as dizziness, vomiting, poor sleep, allergies, and even liver damage. Animal studies have shown that the use of antibiotics such as ENRO may increase the severity of collagen-induced arthritis [3]. Different nations or organizations have established maximum residue limits (MRLs) for ENRO. MRLs for drug-active substances in food are set at 100 μg/kg in bovine, ovine, caprine, and poultry (muscle and fat) by European Commission Regulation (EU) No. 37/2010, which was published in 2009 [4], respectively. However, ENRO is still used excessively by illegal traders in the farming of livestock and aquatic products. Therefore, it is essential to develop a method that enables rapid and accurate detection of ENRO in food matrices to strictly regulate the use of ENRO.

Currently, ENRO detection methods include chemiluminescence immunoassay [5], sensor method [6,7], fluorescence immunoassay [8], colloidal gold immunochromatography [9], and high-performance liquid chromatography (HPLC) [10]. However, the accuracy, precision, rapidity, and sensitivity of these methods are always difficult to balance. The HPLC method requires tedious sample pretreatment and expensive equipment. Colloidal gold immunochromatography is less sensitive, while fluorescence immunoassay results cannot be stored for a long time due to photobleaching. Chemiluminescence immunoassay is affected by environmental factors and its detection accuracy is not ideal. As a result, it is necessary to establish a high-precision, low-cost, and highly selective method applicable to the detection of ENRO remnants in large quantities. To reduce the illegal use of this chemical, a quick and extremely sensitive analytical method for ENRO in meat products must be established.

Surface-enhanced Raman spectroscopy (SERS) has been extensively studied in the area of food safety [11]. With the advantages of rapid detection, small sample size, anti-water interference, and high sensitivity, SERS has been widely employed to detect hazardous food additives [12,13], pathogenic microorganisms [14,15], biotoxins [16,17,18], and pesticide residues [19,20]. Because of its extremely high sensitivity (up to 10^14^), the SERS technique has great potential for single-molecule analysis [21] and detection of unknown objects.

Currently, there are two explanations for the mechanism of this signal enhancement: electromagnetic enhancement and chemical enhancement. The electromagnetic enhancement factor is in the order of 10^5^–10^7^, whereas the contribution of the chemical enhancement factor is only in the order of 10^1^–10^3^ [22]. Localized surface plasmon resonance (LSPR) is responsible for electromagnetic field enhancement and is the main contribution to SERS enhancement [23], and LSPR-based biosensors have enabled the sensitive detection of creatinine [24], cardiac troponin I [25], alanine aminotransferase [26], cholesterol [27], and other biomolecules. Moreover, an important condition for the generation of the SERS effect is the synthesis of rough metal surfaces, mostly using noble metals such as gold, silver, and copper as active substrates because of their excellent electron transport capabilities [28]. Gold and silver nanomaterials have been widely used as SERS active substrates [29,30], and the unique properties of semiconductor nanomaterials among non-metallic nanomaterials make them very valuable SERS substrate materials [31]. In general, gold nanoparticles (AuNPs) have better stability and silver nanoparticles (Ag NPs) have higher SERS activity. Ag NPs are especially more popular in SERS detection, usually with a size smaller than 100 nm [32]. Ag NPs have received increasing attention due to their perfect morphological controllability, sensitive signals, and good chemical stability [33]. Since mammalian or microbial cells can also be killed as a result of the free release of silver ions from Ag NPs, Ag NPs are considered broad-spectrum antimicrobial agents. [34].

However, there is a high demand to combine techniques that are interference-resistant and selective to improve the assay sensitivity and accuracy due to the complexity of food matrices and the susceptibility of detection results to interference from other components. Depending on the scope of application, molecularly imprinted polymers (MIPs) can be prepared to meet different needs by using functional monomers, template molecules, initiators, and crosslinkers in a polymerization reaction. The template molecules in MIPs can be eluted to obtain imprinted cavities that match the size, shape, and functional groups of the template molecules, and such polymers can selectively recombine with the template molecules from substances with similar structures by selective adsorption [35]. As MIPs do not require rigid pH and temperature settings and have better applicability, they have been extensively employed for the detection of antibiotic residues [36]. Xie et al. developed a SERS nano-sensor combined with molecular imprinting technology and synthesized a stable and repeatable composite substrate Cap-MIPs-Au using chloramphenicol (CAP) as a template for the detection of CAP residue in food, with a detection limit of 1 × 10^−7^ g/mL and a recovery rate of 96.6–97.9% [37]. To significantly boost the sensitivity of this MIPs-SERS sensor, Carrasco et al. developed a label-free composite nano-sensor for the detection of residual ENRO in foods that were based on SERS and employed the RAFT method in reactive controlled radical polymerization (LCRP) [38]. However, immobilizing the LCRP reagent on the surface of the substrate remains challenging and has a low success rate.

**Figure 1:**
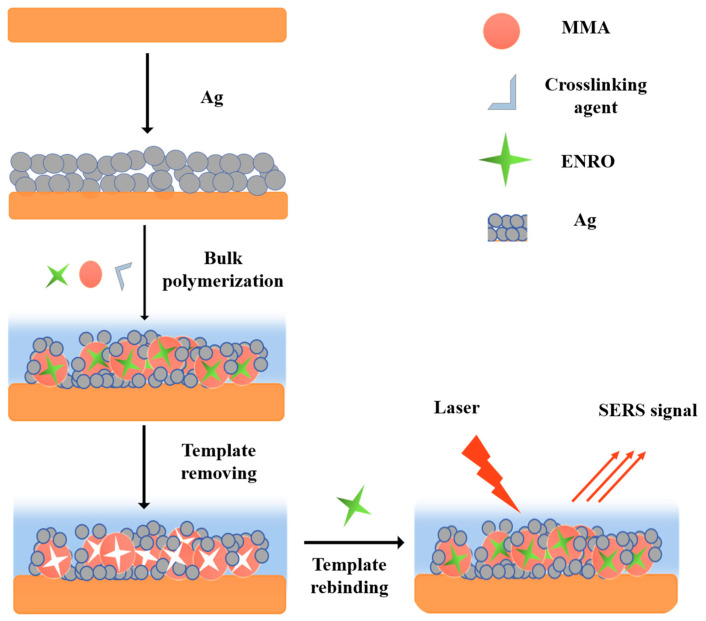
Preparation and recognition schematic diagram of Ag NPs–MIPs and SERS detection in ENRO.

The main objective of this study was to use the SERS technique in conjunction with MIPs to achieve the desired detection sensitivity and accuracy. In SERS detection, Ag NPs were selected as the SERS-enhanced materials because they exhibited perfect shape controllability, sensitive signals, and good bioactivity. To improve the affinity and selectivity of target molecules in close proximity to the surface of noble metals and to achieve selective adsorption of target substances based on the specificity of MIPs, Ag NPs with the SERS effect combined with MIPs with a specific recognition effect were used as substrates. MIPs targeting ENRO were created by native polymerization using MMA as the functional monomer, dimethyl acrylate 1, 4-butylene glycol ester as a crosslinker, phenyl silane as an initiator, and ENRO as the template molecule, as shown in Figure 1. The molecularly imprinted polymer was able to selectively detect and absorb ENRO in complicated food matrices and could be reused after the ENRO template was removed following the completion of polymerization. Finally, SERS was employed to detect and quantify ENRO in MIPs.

In this work, the synthesis and characterization of Ag NP layers and MIPs, the detection limits of ENRO, and the corresponding linear ranges were investigated. Additionally, by contrasting ENRO with structural analogues such as ofloxacin, the exclusive selective detection ability of ENRO was assessed. The accuracy of this method for detecting ENRO residues in pork was also confirmed by comparing the results to those from HPLC. It offers a potential ultra-sensitive and selective platform for the MIPs–SERS-based detection of ENRO residues in food.

The scheme proposed in this paper has the following advantages: (1) A method combining MIPs and SERS techniques is explored, a MIPs film with high selectivity is synthesized, and this MIPs film is used as a SERS substrate to integrate sample pretreatment and rapid detection, establishing a highly selective, low-cost, and error-free detection technique. (2) The MIPs are wrapped with a Ag NPs layer, which can avoid the oxidation process of Ag NPs and better retain the properties of noble metals. (3) Ag NPs can be produced in a simple, quick, reliable, green, and non-toxic manner, and under optimal conditions, they can be obtained in large quantities with diverse morphologies. (4) The template molecules are easily separated from the MIPs and can be reused, which is in line with the concept of green and sustainable chemistry.

## 2. Materials and Methods

### 2.1. Material and Instruments

The following materials were purchased from Shanghai Aladdin Bio-Chem Technology Co., Ltd. (Shanghai, China): trifluoroacetic acid silver salt (98%), phenyl silane (99%), ENRO (95%), methyl methacrylate (AR), 1,4-butanediol dimethacrylate (95%), ciprofloxacin (99.5%), and acetone (99.9%). Methanol, acetic acid, and tetrahydrofuran were purchased from Shanghai Lingfeng Chemical Reagent Co., Ltd. (Shanghai, China). Moxifloxacin (98%) was obtained from Shanghai Bide Pharmaceutical Technology Co., Ltd. (Shanghai, China). Ethanol (95%) was obtained from Sinopharm Chemical Reagent Co., Ltd. (Shanghai, China). From Shanghai Maclean Biochemical Technology Co., Ltd. (Shanghai, China), norfloxacin (98%) was purchased. Copper rods (99.9%) were acquired from Shanghai Daoguan Rubber & Plastic Hardware Co. Ltd. (Shanghai, China).

Using a Zeiss Gemini G500 (Carl Zeiss AG., Oberkochen, Germany), scanning electron microscope, and an accelerating voltage of 5 kV, field emission scanning electron microscopy was used to examine the surface morphological structures of the samples at various magnifications. Energy-dispersive X-ray spectrometry (EDX) was used to examine the composition and elemental distribution of the prepared samples.

With the help of a DeltaNu 785 Raman spectrometer (DeltaNu Inc., Laramie, WY, USA), SERS spectra were gathered. The spectrometer has a spectral range of 200 to 2000 cm^−1^, a laser power of 120 mW, and an excitation wavelength of 785 nm. The spectra were collected with baseline off using the GRAMS/AI (V er 9.1, Thermo Fischer Scientific, Waltham, MA, USA) software and NuSpec software (Copyright DeltaNu 2009).

Using a 752N UV-Visible spectrophotometer (Thermo Fisher scientific, Madison, WI, USA), UV absorption spectra were obtained by adding aqueous solutions of stripped Ag NPs, MIPs, and Ag NPs–MIPs in a quartz cuvette at room temperature.

Using Cu Kα radiation (λ = 0.1541 nm) and a PANalytical X’Pert PRO powder diffractometer (X-ray diffractometer, PANalytical, The Netherlands), X-ray diffraction (XRD) patterns were captured. The working voltage and current were 40 kV and 40 mA, respectively. The patterns were collected with a 2θ range from 0° to 90° at a step of 0.0167°.

### 2.2. Preparation of Ag NP Substrates

A silver film was synthesized on the surface of copper rods by in situ reductions. The 5 cm copper rods (1.0 mm in diameter) were cut and polished smoothly with 600-grit sandpaper then placed into a 5 mL centrifuge tube and washed successively in distilled water, ethanol, acetone, and tetrahydrofuran solution for 5 min by ultrasonication to remove the impurities and oxides on the surface. Afterward, the washed copper rods were immersed in clean tetrahydrofuran solution and stored for backup. Prepared 2.0 mL aqueous silver trifluoroacetate solution with a 10.0 mg/mL concentration was added to a 5 mL centrifuge tube that was kept in a constant 30 °C temperature on a digital thermostat heating plate, followed by heating for 15 min to a constant temperature. After the solution was kept at a constant temperature, the treated copper rods were dried and put into the reaction tube for 5 min; after the reaction was finished, the copper rods were carefully removed with tweezers. Distilled water and ethanol were used to wash away any remaining silver trifluoroacetate solution from the surface. After the reaction was stopped, the silver film-covered copper rods were placed at room temperature to dry naturally.

### 2.3. Preparation of Template-Containing MIPs and Template-Free NIPs

The Ag NPs–MIPs containing template molecules wrapped on the surface of Ag NP films (Ag NPs–MIPs) were prepared as follows: a 5 mL reaction tube was filled with 1.5 mL of MMA monomer that had been purified by an alkaline alumina column to get rid of the inhibitor, followed by the addition of 100 μL of 1, 4-butanediol dimethacrylate cross-linker and 5.0 mg of ENRO, which was fully dissolved by ultrasonication for 1 min, followed by de-oxygenation by high-purity nitrogen (N_2_) for 6 min. The copper rods with the silver film attached were infiltrated with a layer of phenyl silane, then immediately put into the reaction tube and sealed. After that, a copper rod covered in synthesized Ag NPs–MIPs was obtained by conducting a 1-h polymerization reaction at a constant temperature of 75 °C.

The imprinted polymer attached to the copper rod was placed in a 5 mL centrifuge tube. Briefly, 10% methanol acetate solution was added as the elution solvent and eluted in a shaker (300 r/min) for 3 h. The elution solution was changed every 20 min during the elution process. A Raman spectrometer was used to confirm the complete elution and removal of ENRO molecules.

Ag NPs with non-imprinted polymers (NIPs) were fabricated based on the same approach in the absence of ENRO as a blank parallel addition.

### 2.4. Desorption and Regeneration Experiments of Ag NPs–MIPs

The synthesized Ag NPs–MIPs were put into 10% methanolic acetate solution and eluted in a shaker (300 r/min) for 3 h to complete the desorption of ENRO. The regenerated Ag NPs–MIPs were removed and could be reused as ENRO adsorption.

### 2.5. Detection of ENRO Using Ag NPs–MIPs

Dynamic adsorption experiments were used to determine the binding equilibrium time of Ag NPs–MIPs and NIPs. Briefly, 8 eluted MIPs and NIPs polymers were immersed in 2 mL of ENRO solution at a concentration of 1.0 g/mL. The Ag NPs–MIPs and NIPs in one adsorption assay were removed at intervals of every 20 min between 20 and 160 min to stop the adsorption and washed three times with 10% methanol acetate solution to remove the excessive ENRO from their surfaces. Following natural drying, 10 distinct places of the polymers were chosen, and they were each laser-interrogated for 30 s of integration time. To obtain the average value, each experiment was conducted in triplicate. The peak intensity at 1629 cm^−1^ which was detected using a Raman spectrometer was plotted against the adsorption time.

In the resorption study of ENRO, standard solutions of ENRO at concentrations of 0.001 μg/mL, 0.005 μg/mL, 0.015 μg/mL, 0.03 μg/mL, 0.05 μg/mL, and 0.1 μg/mL were made, and 10 different positions of the polymer film were selected after adsorption with Ag NPs–MIPs. In addition, the Raman spectrometer with a high laser intensity measured the peak intensity at 1629 cm^−1^. After three sets of parallel experiments with an integration time of 30 s/time, the average value was determined. The linear equation and correlation coefficient were obtained by plotting the logarithmic peak intensity at 1629 cm^−1^ against the logarithmic concentration of ENRO.

The detection process of ENRO using silver nanomembrane-bound NIPs as substrates was the same as above (except that the MIPs were replaced by NIPs).

### 2.6. Ag NPs–MIPs for Food Applications

The samples of pork used in this study (Shuanghui Group, China) were purchased from a local shop in Hangzhou, Zhejiang, China. ENRO was not detected in the original, purchased samples.

Pork samples were pulverized into minced pork by a pulverizer at 650W and 25,000 r/min. Briefly, in 50 mL centrifuge tubes, 3 portions of the 5.0 g minced pork samples were precisely weighed, and they were then combined with 1.0 mL of ENRO solution at concentrations of 0.1 μg/mL, 0.05 μg/mL, and 0.02 μg/mL, respectively, and stayed still for 15 min to allow ENRO to be absorbed into the minced pork. The supernatant of ENRO extracted from pork was collected twice by adding 10 mL of methanol to each tube and then sonicated for 3 min. Afterward, a low-speed centrifuge was used to spin the sample at 8000 rpm for 8 min. The supernatant was mixed and concentrated to 1–3 mL using rotary evaporation and then concentrated using nitrogen blowing and fixed to 1.0 mL. The concentrated extracts were then adsorbed with the Ag NPs–MIPs prepared in Section 2.4 that specifically identify ENRO for 120 min. Samples spiked with ENRO were processed and assayed in accordance with the steps outlined in Section 2.5.

After the adsorption was completed, the extracts were washed off from the surface of Ag NPs–MIPs with water and dried, detected by Raman spectroscopy. To determine the sample recovery and relative standard deviation (RSD), three parallel experiments were run for each ground pork sample that had been spiked with ENRO.

### 2.7. Determination of ENRO in Food by HPLC

ENRO residues in pork were analyzed by high-performance liquid chromatography (HPLC) in accordance with the Ministry of Agriculture Standard 1025 Notice 14-2008 in order to evaluate the precision of the MIPs–SERS method for detecting ENRO in pork.

The standard solutions of ENRO with concentration gradients of 0.01 μg/mL, 0.02 μg/mL, 0.03 μg/mL, 0.05 μg/mL, and 0.1 μg/mL were prepared for HPLC analysis, and the linear equation and correlation coefficient were acquired by plotting the logarithmic concentration of ENRO against the logarithmic peak area of ENRO after fitting. The pretreatment and spiking methods of pork samples were the same as the process described in Section 2.6, and the extracts were purified after spin concentration. The concentrated extract was run through the C18 solid phase extraction column after it had been pre-washed with 3 mL of methanol and phosphate buffer. It was then eluted with 1.0 mL of purified water and squeezed dry. Afterward, 1.0 mL of mobile phase was eluted, squeezed dry, and the eluate was injected into a liquid phase injection vial after being collected and filtered through a 0.22 μm film for measurement. The HPLC conditions were as follows: C_18_ column (4.6 × 250 mm × 4 μm); mobile phase A injection volume: 10% acetonitrile; mobile phase B injection volume: 90% of 0.1% formic acid aqueous solution; flow rate: 1 mL/min; detection wavelength: 280 nm; column temperature: 30 °C; injection volume: 20 μL.

## 3. Results

### 3.1. Characterization of the Ag NPs

The Ag NPs layer had a simple and time-saving preparation process, and Ag NPs had superior SERS activity than Au NPs, which made them an ideal SERS substrate [39]. In this study, a silver film was synthesized on the surface of copper rods by the reduction method.

**Figure 2:**
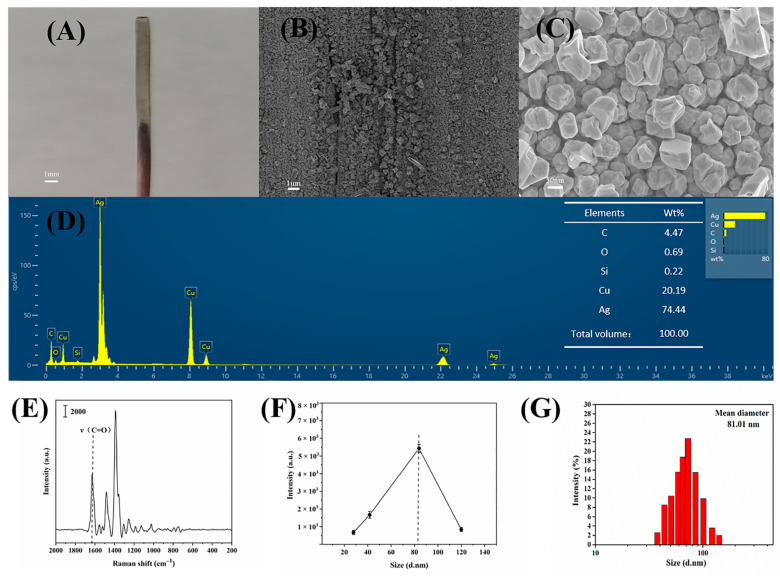
(**A**) Surface appearance of copper rod coated with Ag NPs layer. (**B**) Surface appearance of Ag NPs layer. (**C**) Morphology of Ag NPs. (**D**) Energy-dispersive X-ray (EDX) spectra of Ag NPs. (**E**) SERS spectra of silver films as a substrate for detection of ENRO. (**F**) Effect of particle size on SERS effect. (**G**) The particle size distribution of Ag NPs.

Figure 2A shows the copper rod after the reaction. It could be observed that a silver-black silver film layer was generated on the surface of the purple-red copper rod. The bottom layer was a dense silver-gray film, the surface of which was covered with nearly round Ag NPs stacked into a loosely porous structure.

Figure 2B shows the surface view of the Ag NPs film under SEM. The surface of the silver film was uneven, and the Ag NPs were near the sphere with an unsmooth surface. The SERS enhancement was very strong due to the narrow gap formed between adjacent nanoparticles, and this morphology of Ag NPs had been proved to be a good SERS substrate [40,41].

Figure 2C shows the surface morphology of Ag NPs under SEM. The particle size of Ag NPs ranges from a few tens to a hundred nanometers. The Ag NPs were relatively close to each other, and some of the particles with large particle sizes started to contact each other to form concave spaces so that the incident light scattered several times at the particle gaps, which favored the development of a stronger “hot spot effect” [42]. The “hot spot effect” is mainly generated at the sharp angles where the electromagnetic field distribution is concentrated, which can greatly enhance the intensity of scattered light [43].

Figure 2D shows EDX plots of Ag NPs, C, Cu, Si, and Ag, where C was mainly from the ultrathin supporting carbon film, Cu from the copper mesh skeleton, Si from the resin fixative in the ultrathin section, and Ag from the synthesized silver film.

Figure 2E shows the SERS spectra after the adsorption of 1 mg/mL ENRO soaked with Ag NPs. According to the characteristic peaks at 1629 cm^−1^ and 1468 cm^−1^, which correspond to the C=O stretching vibration peaks of ENRO, the surface-mounted Ag NPs in the silver film had a good enhancing effect on the Raman signal. Since the irregularly shaped Ag NPs had many “inflection points” on their surface where the charge was relatively concentrated, a strong electric field was formed locally when the laser irradiated, and the strong electromagnetic field exhibited localized surface plasmon resonance and strong coupling with the analyte molecules, thus exhibiting a highly enhanced SERS signal of the analyte molecules at their edges. The substrate obtained a highly intense SERS enhancement effect, which enabled sensitive detection of ENRO.

Figure 2F shows the effect of Ag NPs’ particle size on the SERS intensity of the ENRO characteristic peak at 1629 cm^−1^.

Figure 2G indicates that the average particle size of Ag NPs of 81.01 nm obtained under optimal conditions was when Ag NPs had the best SERS enhancement effect on ENRO molecules.

### 3.2. Characterization of the Template-Containing Ag NPs–MIPs

SERS technology was combined with Ag NPs–MIPs by attaching a molecularly imprinted polymer film with the SERS enhancement effect on the surface of the silver film; to be specific, Ag NPs–MIPs films that can specifically recognize ENRO were synthesized as SERS substrates.

The synthetic device for developing the MIPs–SERS sensor, the prepared Ag NPs–MIPs, and the SERS spectral collection device are shown in Figure S1 (Supplementary Materials).

**Figure 3:**
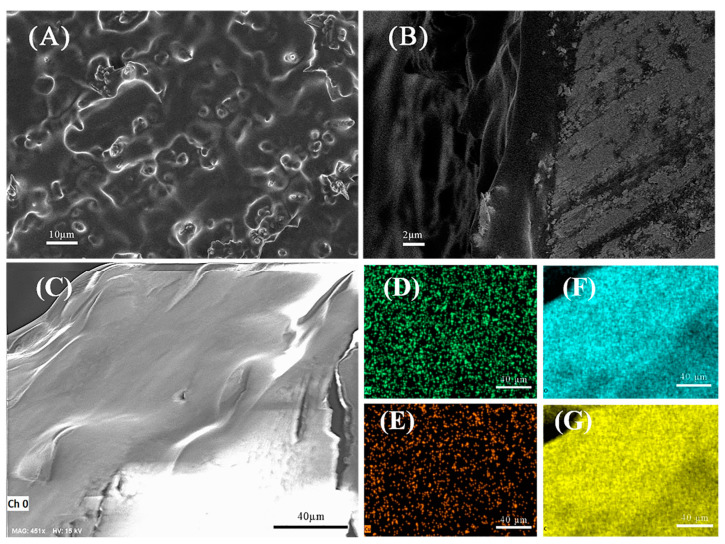
(**A**) Surface of Ag NPs–MIPs film. (**B**) Cross-section of Ag NPs–MIPs film. (**C**) EDX images of the surface and the composition of silver (**D**), copper (**E**), oxygen (**F**), and carbon (**G**) of the Ag NPs–MIPs films.

The SEM images of Figure 3A show that the Ag NPs–MIPs film prepared by this method was uniformly covered on the surface of Ag NPs so that the surface smaller grooves were covered.

Figure 3B shows the cross-section of the Ag NPs–MIPs film where the polymer layer and the Ag NPs layer were tightly polymerized together, and the Ag NPs were also in a stable state in the Ag NPs–MIPs film. According to the classical distance-dependent excited Raman scattering mechanism, the thinner the thickness of Ag NPs–MIPs, the greater the surface enhancement effect [44,45].

The EDX mapping technique was used to determine the elemental distribution of molecularly imprinted polymers. In the region of Figure 3C, there were silver from Ag NPs (Figure 3D), copper from copper rods (Figure 3E), and oxygen (Figure 3F) and carbon (Figure 3G) from molecularly imprinted polymers, thus clearly indicating that a large quantity of Ag NPs were distributed in molecularly imprinted polymers, which demonstrates that the Ag NPs–MIPs could be employed as a SERS active substrate.

In Figure S2, the UV absorbance spectra of Ag NPs, MIPs, and Ag NPs–MIPs in the three synthesis stages are shown. The absorption intensity at 415 nm increases significantly due to the presence of a large number of silver nanoparticles on the sensor surface, which can be attributed to the LSPR absorption of silver nanoparticles. The absorption peak of silver nanoparticles was not detected in pure MIPs. When Ag NPs were wrapped by MIPs, the absorption intensity slightly decreased. This can prove that the Ag NPs–MIPs synthesized in the system do contain Ag NPs, which is a prerequisite for achieving the SERS enhancement effect.

### 3.3. SERS Selectivity and Reusability of Ag NPs–MIPs

MIPs provided a large number of functional groups and imprinted cavities to specifically recognize ENRO molecules and specifically adsorb them to the surface of Ag NPs. The ability of MIPs to recognize and rebind ENRO depended on the adsorption time. If the adsorption time was too short, the Ag NPs–MIPs could not fully recognize and bind to ENRO in the environment. If the adsorption time was too long, the effect of the experiment was greatly reduced. Determining the ideal adsorption time was essential.

**Figure 4:**
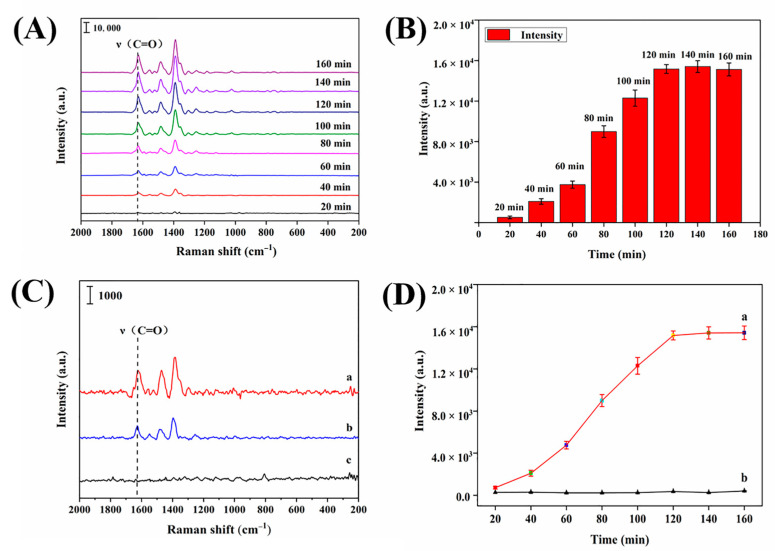
(**A**) SERS spectra obtained at different adsorption times for 1 μg/mL ENRO absorbed on Ag NPs–MIPs film as substrate. (**B**) Effect of adsorption time on the SERS intensity of ENRO. (**C**) SERS spectra of 1 μg/mL ENRO absorbed by Ag NPs film as substrate (a), 1 μg/mL ENRO absorbed by Ag NPs–MIPs as substrate (b), and 1 μg/mL ENRO absorbed by NIPs as substrate (c). (**D**) Kinetic adsorption assay of Ag NPs–MIPs film (a), kinetic adsorption assay of NIPs film (b).

As seen in Figure 4A, the peak intensity at 1629 cm^−1^ gradually increased with the adsorption time, and after 120 min, the peak intensity stopped increasing noticeably.

According to Figure 4B, the peak intensity increased more gradually between 20 and 60 min after adsorption. At 60~120 min, the intensity peaked out significantly. After 120 min of adsorption, the peak intensity remained largely stable, indicating that the adsorption of ENRO by Ag NPs–MIPs had reached saturation and 120 min was the optimal equilibrium time.

To verify the ability of the synthesized Ag NPs–MIPs film to specifically adsorb ENRO molecules, it was compared with the NIPs film without an ENRO template. According to Figure 4C, a distinct ENRO characteristic peak could be seen on the spectra of ENRO detection with the Ag NPs film and Ag NPs–MIPs film as the SERS substrate; in contrast, no ENRO characteristic peak appeared on the spectra of ENRO detection with the NIPs film.

Figure 4D’s kinetic adsorption experiments indicated that Ag NPs–MIPs had superior adsorption efficiency and reach equilibrium around 120 min. The adsorption capacity of Ag NPs–MIPs on ENRO was significantly stronger than that of NIPs, indicating that a large number of imprinted cavities were generated after template removal.

The above results confirmed that the Ag NPs–MIPs film fabricated by this method contains imprinted cavities that match the structure of ENRO molecules and can selectively adsorb ENRO molecules, whereas the NIPs film was bound to ENRO molecules mainly by physical adsorption and was not able to selectively adsorb ENRO molecules.

To explore the reusable performance of the Ag NPs–MIPs synthesized by this method, we regenerated the Ag NPs–MIPs by eluting them with 10% methanol acetate solution, and the results are shown in Figure S3. The adsorption capacity of Ag NPs–MIPs started to decrease slightly after four washes, which was mainly due to the fact that a small number of template molecules were harder to elute in the interlayer of Ag NPs–MIPs, and a small number of blot cavities were destroyed during the elution. However, after ten elutions, the overall adsorption capacity could still be maintained at a high level, which confirmed the good reusability of Ag NPs–MIPs as SERS sensors and laid the foundation for practical applications.

### 3.4. Specificity of the Ag NPs–MIPs

**Figure 5:**
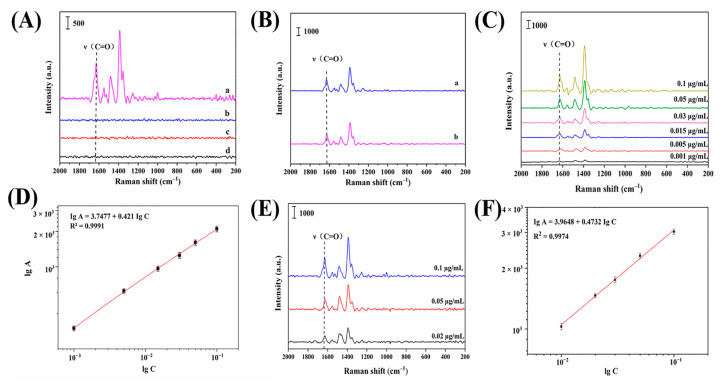
(**A**) SERS spectra of Ag NPs–MIPs film adsorption of 1.0 µg/mL ENRO (a), SERS spectra of Ag NPs–MIPs film adsorption of 1.0 µg/mL ciprofloxacin (b), SERS spectra of Ag NPs–MIPs film adsorption of 1.0 µg/mL moxifloxacin (c), and SERS spectra of Ag NPs–MIPs film adsorption of 1.0 µg/mL norfloxacin (d). (**B**) SERS spectra of Ag NPs–MIPs film for 1.0 µg/mL ENRO (a), SERS spectra of Ag NPs–MIPs film for 1.0 µg/mL mixed solution containing ENRO, ciprofloxacin, moxifloxacin, and norfloxacin. (**C**) SERS spectra of Ag NPs–MIPs film detection of standard solutions of different concentrations of ENRO. (**D**) Calibration curves of Ag NPs–MIPs film detection of ENRO solutions. (**E**) SERS spectra of Ag NPs–MIPs film adsorption spiked with ENRO pork. (**F**) Calibration curve of ENRO detection by HPLC.

ENRO, ciprofloxacin, moxifloxacin, and norfloxacin all belong to quinolone antibiotics, which have similar molecular structures. To further verify the specific adsorption of Ag NPs–MIPs on ENRO molecules in this study, Ag NPs–MIPs were adsorbed with 1.0 µg/mL of ENRO, ciprofloxacin, moxifloxacin, norfloxacin, and mixed solutions for SERS assay, respectively, which are shown in Figure 5.

As shown in Figure 5A, the Ag NPs–MIPs showed obvious ENRO characteristic peak signals in the SERS spectra after adsorption in ENRO solution for some time. Meanwhile, the SERS spectra of the Ag NPs–MIPs adsorbed with ciprofloxacin, moxifloxacin, and norfloxacin solutions did not exhibit the recognizable peak signals of the corresponding substances.

As shown in Figure 5B, the Ag NPs–MIPs that adsorbed the mixed solutions of the four substances showed obvious ENRO characteristic peak signals in the SERS spectra, which matched the characteristic peaks of the Raman spectra of the ENRO standard. These experimental results suggested that the ENRO template molecules can be detached from Ag NPs–MIPs, leaving an imprinted cavity matching the ENRO molecules for specific rebinding to the target molecules. When ENRO molecules were present in the detection liquid, the Ag NPs–MIPs could selectively adsorb ENRO molecules into the cavities, thus detecting the SERS signal of ENRO when the Ag NPs–MIPs were used as the SERS substrate. Even if the analogues were present in the cavities that could not match their functional groups or spatial arrangement order, they did not bind firmly in the cavities of the binding sites of Ag NPs–MIPs. Therefore, the Ag NPs–MIPs made using this experimental technique have the capacity to recognize and adsorb ENRO molecules in complex environments.

### 3.5. Detection of ENRO by the Ag NPs–MIPs

The calibration curves were obtained by laser probing standard solutions of ENRO at concentrations ranging from 0.001~0.1 μg/mL under optimal experimental conditions.

The SERS spectra of the ENRO solutions measured at various concentrations are displayed in Figure 5C. Peak intensity at 1629 cm^−1^ was observed to increase gradually as ENRO concentration increased from 0.001 to 0.1 μg/mL.

As shown in Figure 5D, in the concentration range of 0.001~0.1 μg/mL, the logarithm of the concentration of ENRO standard solution (lg C) displayed a satisfied linear relationship with the logarithm of the intensity of the characteristic peak at 1629 cm^−1^ (lg A). The linear equation was Ig A = 3.7477 + 0.421 Ig C (R^2^ = 0.9991). The limit of detection (LOD) of this assay was 0.025 ng/mL. The recoveries of 96.73% to 99.41% with RSDs of 1.9% to 6.22% are demonstrated in Table S1. The accuracy of the calibration curve for the detection of ENRO as a substrate was good, and it can be used as a quantitative analytical assay for ENRO.

### 3.6. Detection of ENRO by the Ag NPs–MIPs for Spiked Food

The Ag NPs–MIPs were then used to detect ENRO that had been spiked in pork (Shuanghui Cold Fresh, Hangzhou, China).

As can be seen in Figure 5E, the ENRO characteristic peak emerged significantly at wave number 1629 cm^−1^, which indicated that ENRO could be effectively detected after the sample was spiked with ENRO. For the spiking experiments with different concentrations, the intensity of the characteristic peak at 1629 cm^−1^ intensified with increasing concentration; therefore, it can be determined that the MIPs–SERS approach is applicable for the qualitative detection of ENRO in pork.

In order to accurately quantify the content of ENRO in pork spiked with ENRO, three sets of parallel experiments were performed for each concentration and a blank group was set up. In Table S2, the recoveries of pork samples spiked with ENRO by Ag NPs–MIPs as the SERS substrate were 90.61~92.72% with the RSD of 2.42%~5.16%. The good recoveries and precision indicated that the Ag NPs–MIPs film is a robust tool for the detection of ENRO in actual samples.

### 3.7. HPLC Analysis of ENRO in Real Samples

To further confirm the validity of the assay results for the detection of ENRO in food matrices using Ag NPs–MIPs as SERS substrates, this experiment was performed on the basis of the Chinese Ministry of Agriculture Standard 1025 Bulletin-14-2008 with improved liquid chromatographic conditions, and quantitative analysis was performed in pork spiked with ENRO using HPLC. The results were compared with those from the MIPs–SERS assay.

Figure 5F demonstrates a satisfying linear relationship between the logarithm of the concentration (lg C) and the logarithm of the peak area (lg A) in the concentration range of 0.01–0.1 µg/mL for ENRO detection by HPLC, and the fitted linear equation was lg A = 3.9648 + 0.4732 lg C with the correlation coefficient R^2^ = 0.9974.

As shown in Table S3, the experimental results showed that the recoveries of the spiked ENRO samples in pork using HPLC ranged from 96.68% to 101.4% with RSD of 1.62% to 6.18%. By comparing MIPs–SERS with HPLC, the results indicate that the experimental results obtained using the MIPs–SERS approach were consistent with those obtained using the HPLC method, which proves the dependability of Ag NPs–MIPs for the quantitative detection of ENRO in pork.

Overall, the developed MIPs–SERS approach is rapid and does not require time-consuming sample pre-treatment, simplifying experimental processing steps and lowering detection costs. It could recognize and adsorb ENRO in complex food matrices.

Table 1 compares the MIPs–SERS and HPLC methods used in this work with other methods that have been reported for the detection of enrofloxacin. By comparing the response range, LOD, spiked recovery rate, and RSD, it demonstrates that the Ag NPs–MIPs film prepared in this work had good detection sensitivity and is applicable for the detection of ENRO in real samples.

## 4. Conclusions

In this study, the Ag NPs–MIPs with high selectivity was prepared. Firstly, a Ag NPs film with excellent SERS performance was synthesized on the surface of a copper rod by chemical reduction, and the mechanism of the SERS enhancement effect of Ag NPs on the surface of the silver film was investigated, and the silver film with the optimal SERS enhancement effect was prepared by optimization of the experimental formulation and reaction conditions. By combining the SERS technique with the MIT technique, the established MIP–SERS method using Ag NPs–MIPs as the SERS substrate keeps the benefits of the high sensitivity of SERS while enhancing the ability to adsorb target compounds in complicated sample matrices by employing MIPs. The method can reduce detection costs because the detection process only requires a small amount of samples. Rapid and on-site detection is also achieved by introducing a portable Raman spectrometer.

Additionally, the accuracy and dependability of the MIPs–SERS approach for the qualitative and quantitative determination of ENRO were confirmed by a comparison with the assay results of the HPLC method.

The proposed MIPs–SERS method provides a time-saving, cost-effective, highly sensitive and selective tool which can also be further enhanced for the quick analysis of other restricted and forbidden compounds to ensure the safety of different food products.

## Figures and Tables

**Table 1:** Comparison of various methods for the detection of residual enrofloxacin.

Methodology	Response Range (μg/mL)	LOD (μg/mL)	Spiked Recovery Rate (%)	RSD (%)	Reference
Fluorescent carbon dots	1~15	0.16	96.5~109	1.5~2.3	[46]
Terbium-sensitized fluorescence	0.05~0.6	0.021	87~97	3.1~15	[47]
Voltammetry	0.025~1.0	0.0057	99.04~106.76	3.72~7.22	[48]
Biomimetic enzyme-linked immunosorbent assay	0.001~10	0.0011	74.4~86.8	4.3~7.9	[49]
Localized surface plasmon resonance	0.025~1	0.0611	80.7~95.4	2.9~7.7	[50]
Bacterial respiration	0.005~1	0.01	—	4.45~4.88	[51]
MIPs–SERS	0.001~0.1	0.00025	90.61~92.72	2.42~5.16	This work
HPLC	0.01~0.1	0.003	96.68~101.4	1.62~6.18	This work

## Data Availability

Raw data presented in this study are available on request from the corresponding author.

## Supplementary Materials

The following supporting information can be downloaded at: https://www.mdpi.com/article/10.3390/bios13030330/s1, Figure S1: Synthesis device for developing MIPs–SERS sensors, Ag NPs–MIPs and SERS spectral collection equipment; Figure S2: UV absorbance spectrum at each stage of Ag NPs, MIPs, Ag NPs–MIPs; Figure S3: Number of regeneration cycles of Ag NPs–MIPs; Table S1: Percentage recoveries and relative standard deviation (RSD) of the detection of ENRO spiked in samples (mean ± SD, N = 3); Table S2: Recovery and precision analysis of detection of spiked ENRO in pork using Ag NPs–MIPs as SERS substrates; Table S3: Recovery and precision analysis of detection of spiked ENRO in pork using HPLC.

## References

[1] Nemeth J., Oesch G., Kuster S.P. (2015). Bacteriostatic versus bactericidal antibiotics for patients with serious bacterial infections: Systematic review and meta-analysis. J. Antimicrob. Chemother..

[2] Blasco C., Picó Y., Torres C.M. (2007). Progress in analysis of residual antibacterials in food. Trac-Trends Anal. Chem..

[3] Dorożyńska I., Majewska-Szczepanik M., Marcińska K., Szczepanik M. (2014). Partial depletion of natural gut flora by antibiotic aggravates collagen induced arthritis (CIA) in mice. Pharmacol. Rep..

[4] (2009). Official Journal of the European Union L 15/1 of 22 December 2009.

[5] Li J., Lu S., Xiang J., Xu X., Wei L., Cheng X. (2019). Class-specific determination of fluoroquinolones based on a novel chemiluminescence system with molecularly imprinted polymers. Food Chem..

[6] Wang M., Hu M., Liu J., Guo C., Peng D., Jia Q., He L., Zhang Z., Du M. (2019). Covalent organic framework-based electrochemical aptasensors for the ultrasensitive detection of antibiotics. Biosens. Bioelectron.

[7] Liu X., Ren J., Su L., Gao X., Tang Y., Ma T., Zhu L., Li J. (2017). Novel hybrid probe based on double recognition of aptamer-molecularly imprinted polymer grafted on upconversion nanoparticles for enrofloxacin sensing. Biosens. Bioelectron..

[8] Hu G., Sheng W., Zhang Y., Wu X., Wang S. (2015). A novel and sensitive fluorescence immunoassay for the detection of fluoroquinolones in animal-derived foods using upconversion nanoparticles as labels. Anal. Bioanal. Chem..

[9] Byzova N.A., Smirnova N.I., Zherdev A.V., Eremin S.A., Shanin I.A., Lei H.T., Sun Y., Dzantiev B.B. (2014). Rapid immunochromatographic assay for ofloxacin in animal original foodstuffs using native antisera labeled by colloidal gold. Talanta.

[10] Yu H., Mu H., Hu Y.M. (2012). Determination of fluoroquinolones, sulfonamides, and tetracyclines multiresidues simultaneously in porcine tissue by MSPD and HPLC-DAD. J. Pharm. Anal..

[11] Cialla D., Marz A., Bohme R., Theil F., Weber K., Schmitt M., Popp J. (2012). Surface-enhanced Raman spectroscopy (SERS): Progress and trends. Anal. Bioanal. Chem..

[12] Li W., Sun Y., Chen W., Song G., Huang M., Xu J., Shi X., Li P. (2022). Hollow RuCu bimetallic nanospheres as emerging SERS substrates for illegal food additives detection. Mater. Lett..

[13] Wu T., Li J., Zheng S., Yu Q., Qi K., Shao Y., Wang C., Tu J., Xiao R. (2022). Magnetic Nanotag-Based Colorimetric/SERS Dual-Readout Immunochromatography for Ultrasensitive Detection of Clenbuterol Hydrochloride and Ractopamine in Food Samples. Biosensors.

[14] Wang C., Wang C., Li J., Tu Z., Gu B., Wang S. (2022). Ultrasensitive and multiplex detection of four pathogenic bacteria on a bi-channel lateral flow immunoassay strip with three-dimensional membrane-like SERS nanostickers. Biosens. Bioelectron..

[15] Wang R., Kim K., Choi N., Wang X., Lee J., Jeon J.H., Rhie G.-e., Choo J. (2018). Highly sensitive detection of high-risk bacterial pathogens using SERS-based lateral flow assay strips. Sen. Actuators B Chem..

[16] Juneja S., Zhang B., Nujhat N., Wang A.X. (2022). Quantitative Sensing of Domoic Acid from Shellfish Using Biological Photonic Crystal Enhanced SERS Substrates. Molecules.

[17] Wang C., Gu B., Liu Q., Pang Y., Xiao R., Wang S. (2018). Combined use of vancomycin-modified Ag-coated magnetic nanoparticles and secondary enhanced nanoparticles for rapid surface-enhanced Raman scattering detection of bacteria. Int. J. Nanomed..

[18] He H., Sun D.W., Pu H., Huang L. (2020). Bridging Fe_3_O_4_@Au nanoflowers and Au@Ag nanospheres with aptamer for ultrasensitive SERS detection of aflatoxin B1. Food Chem..

[19] Alsammarraie F.K., Lin M., Mustapha A., Lin H., Chen X., Chen Y., Wang H., Huang M. (2018). Rapid determination of thiabendazole in juice by SERS coupled with novel gold nanosubstrates. Food Chem..

[20] Hu B., Sun D.W., Pu H., Wei Q. (2020). Rapid nondestructive detection of mixed pesticides residues on fruit surface using SERS combined with self-modeling mixture analysis method. Talanta.

[21] Graham D., Goodacre R. (2008). Chemical and bioanalytical applications of surface enhanced Raman scattering spectroscopy. Chem. Soc. Rev..

[22] Ding S.-Y., Yi J., Li J.-F., Ren B., Wu D.-Y., Panneerselvam R., Tian Z.-Q. (2016). Erratum: Nanostructure-based plasmon-enhanced Raman spectroscopy for surface analysis of materials. Nat. Revi. Mater..

[23] Willets K.A., Van Duyne R.P. (2007). Localized Surface Plasmon Resonance Spectroscopy and Sensing. Annu. Rev. Phys. Chem..

[24] Li M., Singh R., Marques C., Zhang B., Kumar S. (2021). 2D material assisted SMF-MCF-MMF-SMF based LSPR sensor for creatinine detection. Opt. Express..

[25] Wang Y., Singh R., Chaudhary S., Zhang B., Kumar S. (2022). 2-D Nanomaterials Assisted LSPR MPM Optical Fiber Sensor Probe for Cardiac Troponin I Detection. IEEE Trans. Instrum. Meas..

[26] Wang Z., Singh R., Marques C., Jha R., Zhang B., Kumar S. (2021). Taper-in-taper fiber structure-based LSPR sensor for alanine aminotransferase detection. Opt. Express..

[27] Kumar S., Kaushik B.K., Singh R., Chen N.-K., Yang Q.S., Zhang X., Wang W., Zhang B. (2019). LSPR-based cholesterol biosensor using a tapered optical fiber structure. Biomed. Opt. Express.

[28] Zhang L., Miu W.-B., Yao J., Sun L., Yu B. (2018). Magnetic ordered mesoporous carbon composites incorporating Ag nanoparticles as SERS substrate for enrichment and detection of trace mercaptan compounds. Res. Chem. Intermed..

[29] Dutta P., Su T.-Y., Fu A.-Y., Chang M.-C., Guo Y.-J., Tsai I.J., Wei P.-K., Chang Y.-S., Lin C.-Y., Fan Y.-J. (2022). Combining portable solar-powered centrifuge to nanoplasmonic sensing chip with smartphone reader for rheumatoid arthritis detection. Chem. Eng. J..

[30] Yougbaré S., Chou H.-L., Yang C.-H., Krisnawati D.I., Jazidie A., Nuh M., Kuo T.-R. (2021). Facet-dependent gold nanocrystals for effective photothermal killing of bacteria. J. Hazard. Mater..

[31] Okoro G., Husain S., Saukani M., Mutalik C., Yougbaré S., Hsiao Y.-C., Kuo T.-R. (2023). Emerging Trends in Nanomaterials for Photosynthetic Biohybrid Systems. ACS Mater. Lett..

[32] Keat C.L., Aziz A., Eid A.M., Elmarzugi N.A. (2015). Biosynthesis of nanoparticles and silver nanoparticles. Bioresour. Bioprocess..

[33] Mondal S., Rana U., Malik S. (2015). Facile decoration of polyaniline fiber with Ag nanoparticles for recyclable SERS substrate. ACS Appl. Mater. Interfaces.

[34] Vigneswari S., Amelia T.S.M., Hazwan M.H., Mouriya G.K., Bhubalan K., Amirul A.A., Ramakrishna S. (2021). Transformation of Biowaste for Medical Applications: Incorporation of Biologically Derived Silver Nanoparticles as Antimicrobial Coating. Antibiotics.

[35] Fan J., Wei Y., Wang J., Wu C., Shi H. (2009). Study of molecularly imprinted solid-phase extraction of diphenylguanidine and its structural analogs. Anal. Chim. Act..

[36] Li H., Jia X., Jiang W., Zhou T., He J., Luan Y., Shang Y., Liu C., Che G. (2020). Magnetically assisted imprinted sensor for selective detection antibiotics in river based on surface-enhanced Raman scattering. Opt. Mater..

[37] Xie Y., Zhao M., Hu Q., Cheng Y., Guo Y., Qian H., Yao W. (2017). Selective detection of chloramphenicol in milk based on a molecularly imprinted polymer–surface-enhanced Raman spectroscopic nanosensor. J. Raman Spectrosc..

[38] Carrasco S., Benito-Peña E., Navarro-Villoslada F., Langer J., Sanz-Ortiz M.N., Reguera J., Liz-Marzán L.M., Moreno-Bondi M.a.C. (2016). Multibranched Gold–Mesoporous Silica Nanoparticles Coated with a Molecularly Imprinted Polymer for Label-Free Antibiotic Surface-Enhanced Raman Scattering Analysis. Chem. Mater..

[39] Neng J., Zhang Q., Sun P. (2020). Application of surface-enhanced Raman spectroscopy in fast detection of toxic and harmful substances in food. Biosens. Bioelectron..

[40] Ji J., Li P., Sang S., Zhang W., Zhou Z., Yang X., Dong H., Li G., Hu J. (2014). Electrodeposition of Au/Ag bimetallic dendrites assisted by Faradaic AC-electroosmosis flow. AIP Adv..

[41] Kang Y., Wu T., Han X., Gu H., Zhang X. (2018). A needle-like reusable surface-enhanced Raman scattering substrate, and its application to the determination of acetamiprid by combining SERS and thin-layer chromatography. Microchim. Acta.

[42] Aravind P.K., Nitzan A., Metiu H. (1981). The interaction between electromagnetic resonances and its role in spectroscopic studies of molecules adsorbed on colloidal particles or metal spheres. Surf. Sci..

[43] Aravind P.K., Metiu H. (1983). The effects of the interaction between resonances in the electromagnetic response of a sphere-plane structure; applications to surface enhanced spectroscopy. Surf. Sci..

[44] Yang D., Xia L., Zhao H., Hu X., Liu Y., Li J., Wan X. (2011). Preparation and characterization of an ultrathin carbon shell coating a silver core for shell-isolated nanoparticle-enhanced Raman spectroscopy. Chem. Commun..

[45] Li D., Wu S., Wang Q., Wu Y., Peng W., Pan L. (2012). Ag@C Core–Shell Colloidal Nanoparticles Prepared by the Hydrothermal Route and the Low Temperature Heating–Stirring Method and Their Application in Surface Enhanced Raman Scattering. J. Phys. Chem. C.

[46] Guo X., Zhang L., Wang Z., Sun Y., Liu Q., Dong W., Hao A. (2019). Fluorescent carbon dots based sensing system for detection of enrofloxacin in water solutions. Spectroc. Acta. Pt. A-Molec. Biomolec. Spectr..

[47] Ershadi S., Jouyban A., Shayanfar A. (2017). Determination of Enrofloxacin in Milk Samples Using Silver Nanoparticle Enhanced Terbium-Sensitized Fluorescence Method. Food Anal. Meth..

[48] Donmez F., Yardim Y., Senturk Z. (2018). Electroanalytical determination of enrofloxacin based on the enhancement effect of the anionic surfactant at anodically pretreated boron-doped diamond electrode. Diam. Relat. Mat..

[49] Wang J., Sang Y., Liu W., Liang N., Wang X. (2017). The development of a biomimetic enzyme-linked immunosorbent assay based on the molecular imprinting technique for the detection of enrofloxacin in animal-based food. Anal. Methods.

[50] Wang W., Wang R., Liao M., Kidd M.T., Li Y. (2021). Rapid detection of enrofloxacin using a localized surface plasmon resonance sensor based on polydopamine molecular imprinted recognition polymer. J. Food Meas. Charact..

[51] Lee H., Lee S., Kwon D., Yim C., Jeon S. (2017). Microbial respiration-based detection of enrofloxacin in milk using capillary-tube indicators. Sens. Actuato B-Chem..

