# Supplementary material for: MIPs–SERS Sensor Based on Ag NPs Film for Selective Detection of Enrofloxacin in Food
Source: Biosensors (Basel). 2023 Feb 28;13(3):330. doi: 10.3390/bios13030330 (PMC10046510; doi:10.3390/bios13030330)
Supplement: Supplementary file 1 [file biosensors-13-00330-s001.zip › biosensors-2183143-supplementary.pdf]

biosensors MDPI

MIPs-SERS sensor based on Ag NPs film for selective detection of enrofloxacin in food

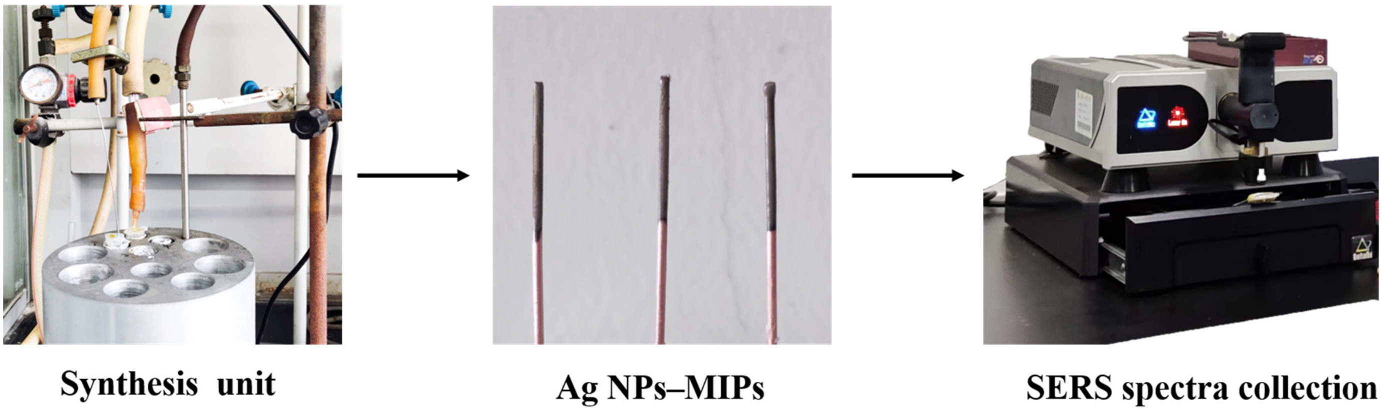

Figure S1. Synthesis device for developing MIPs-SERS sensors, Ag NPs–MIPs and SERS spectral collection equipment

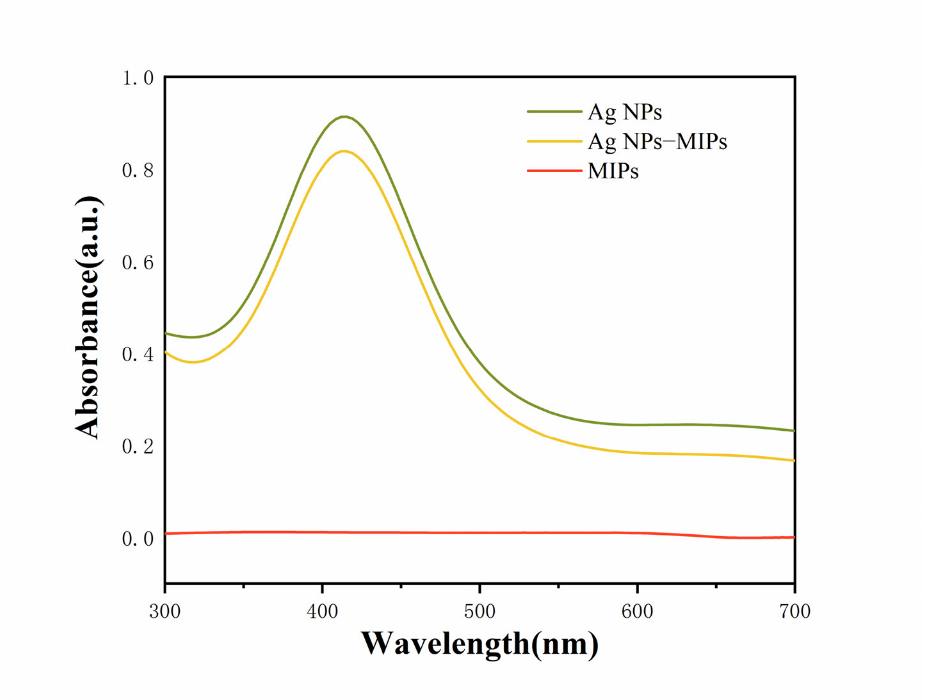

Figure S2. UV absorbance spectrum at each stage of AgNPs , MIPs, Ag NPs–MIPs

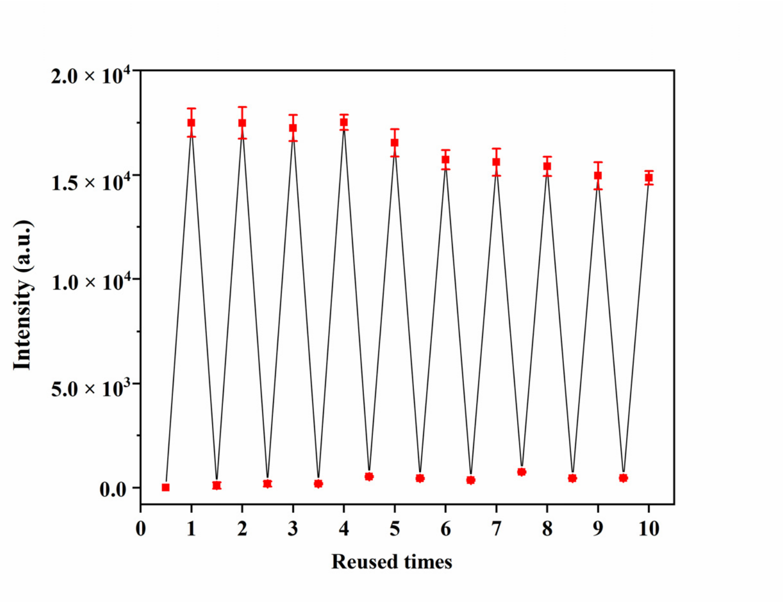

Figure S3. Number of regeneration cycles of Ag NPs–MIPs

Table S1. Percentage recoveries and relative standard deviation (RSD) of the detection of ENRO spiked in samples (mean ± SD, N = 3)

Concentration of ENRO solution	Found ± SD（μg/mL）	Spiked recovery rate（%）	RSD（%）
0.02	0.0199 ± 0.0012	99.41	6.22
0.05	0.0494 ± 0.001	98.74	1.90
0.10	0.0968 ± 0.0031	96.73	3.25

In section 3.5, Table S1 summarizes the recovery and the relative standard deviation (RSD) of the detection of ENRO.

Table S2. Recovery and precision analysis of detection of spiked ENRO in pork using Ag NPs–MIPs as SERS substrates

Pork Sample	Spiked（μg/mL）	Found ± SD（μg/mL）	Spiked recovery rate（%）	RSD（%）
1	0.02	0.0183 ± 0.001	91.70	5.16
2	0.05	0.0494 ± 0.0011	90.61	2.42
3	0.10	0.0927 ± 0.0039	92.72	4.20

In section 3.6, the MIP was further applied to detect ENRO added to pork. the recoveries and RSD values of the samples are shown in Table S2.

Table S3. Recovery and precision analysis of detection of spiked ENRO in pork using HPLC

Pork Sample	Spiked（μg/mL）	Found ± SD（μg/mL）	Spiked recovery rate（%）	RSD（%）
1	0.02	0.0193 ± 0.0012	96.68	6.18
2	0.05	0.0493 ± 0.0008	98.54	1.62
3	0.10	0.1014 ± 0.0027	101.40	2.67

In section 3.7, the recoveries of samples and the values of RSD were demonstrated in Table S3.
